# Proteasome Targeting with Carfilzomib Induces Reactive Oxygen Species-Mediated Apoptosis in Hepatoblastoma

**DOI:** 10.3390/cells15100864

**Published:** 2026-05-09

**Authors:** Elena Johanna Weigl, Salih Demir, Alina Hotes, Emilie Indersie, Sophie Branchereau, Stefano Cairo, Roland Kappler

**Affiliations:** 1 Department of Pediatric Surgery, Dr. von Hauner Children’s Hospital, University Hospital, LMU Munich, Lindwurmstreet 2a, 80337 Munich, Germany; 2 XenTech, 91000 Evry, France; 3 Bicêtre Hospital, AP-HP Paris Saclay University, 94270 Le Kremlin-Bicêtre, France; 4 Champions Oncology, Rockville, MD 20850, USA

**Keywords:** hepatoblastoma, proteasome inhibition, carfilzomib, reactive-oxygen-species pathway

## Abstract

**Highlights:**

**What are the main findings?**

- The proteasome β5 subunit (PSMB5) is overexpressed in hepatoblastoma.
- Proteasome inhibition induces reactive oxygen species signaling and apoptosis in hepatoblastoma models.

**What are the implications of the main findings?**

- PSMB5 may predict response to proteasome inhibition.
- The proteasome inhibitor carfilzomib shows promise as a targeted therapy for hepatoblastoma.

**Abstract:**

Hepatoblastoma (HB) is the most common malignant liver tumor in children, yet therapeutic options remain largely confined to conventional chemotherapy. To identify novel therapeutic targets, we performed gene set enrichment analysis on three publicly available HB datasets and found consistent activation of the proteasome pathway, with marked overexpression of the β5 proteolytic subunit encoded by *PSMB5*. High *PSMB5* expression was associated with poor survival in adult hepatocellular carcinoma, highlighting the proteasome as a candidate for therapeutic vulnerability. Targeting the β5 proteolytic subunit with its selective inhibitor carfilzomib in HB patient-derived xenograft (PDX) models resulted in dose-dependent reductions in cell viability, proliferation, and clonogenic growth, accompanied by induction of apoptosis. Importantly, carfilzomib retained efficacy in three-dimensional PDX cultures, underscoring its activity in physiologically relevant tumor models. Bioinformatic analyses revealed that carfilzomib activates apoptosis and reactive oxygen species (ROS) signaling. Validation experiments in HB cells demonstrated increased ROS levels, with ROS induction correlating with drug sensitivity. Notably, pharmacological scavenging of ROS completely abrogated carfilzomib-induced cytotoxicity, establishing oxidative stress as a key mediator of therapeutic response. Together, these findings identify *PSMB5* as a therapeutically actionable target in HB and support proteasome inhibition as a promising precision medicine strategy in HB.

## 1. Introduction

Hepatoblastoma (HB) is the most frequent malignant liver tumor in children [1,2]. Hepatoblastoma is rare in absolute numbers, with a worldwide incidence of 1–2 cases per million children [3]. While many other tumor entities are now treated using tailored approaches and targeted therapies, HB treatment is still based on conventional chemotherapeutic agents, primarily cisplatin and doxorubicin [4,5]. Overall survival rates are favorable, reaching up to 95% at three years following standard-of-care neo-adjuvant chemotherapy and surgical resection of the tumor [5]. Nevertheless, despite the continuous effort to improve therapy options and treatment protocols, patients with advanced-stage and relapsed HB continue to experience significantly poorer outcomes [4,5].

Targeted therapies are most effective when they focus on a specific molecular alteration [6]. The development of a tailored therapy for HB is hampered by the lack of such a well-defined target. Genetically, HB is a relatively simple tumor entity, with an average of only 2.7 mutations per tumor, making it the pediatric cancer with the lowest reported mutation rate [7]. The most frequent genetic alterations are mutations in *CTNNB1* that activate β-catenin in up to 92% of cases, as well as mutations in *APC* and *AXIN1* [8,9,10,11]. However, during cisplatin treatment, a sudden burst of acquired mutations can occur, leading to chemoresistance and the emergence of patient-specific molecular profiles [10]. A plethora of studies exist, screening the broad genomic landscape of HB in search of new therapeutic targets [8,9,10,11,12,13,14]. Interestingly, newly found core mutations rarely overlap between studies, suggesting that our current understanding captures only fragments of the biology rather than the whole molecular framework of HB [13].

A promising strategy involves identifying non-mutational targets located at the junction of key cellular pathways that control apoptosis, cell growth, DNA repair, cell-cycle checkpoints, and cell division. Targeting such central regulatory nodes could inhibit cancer cell growth via multiple interconnected mechanisms and possibly counteract various mutations simultaneously [6]. One such crossroad of key cellular functions is the proteasome, which is responsible for protein quality control and the degradation of intracellular proteins in eukaryotic cells. It thereby regulates various cellular functions, including cell survival, apoptosis, the cell cycle and cellular metabolism [15,16]. The 26S proteasome is composed of a 20S core particle and two 19S regulatory particles [17,18]. Proteolysis occurs within the barrel-shaped 20S core particle made up of four stacked rings [17]. The inner rings contain proteolytic sites, with β1, β2, and β5 subunits harboring caspase-like, trypsin-like, and chymotrypsin-like activities, respectively [18,19]. These subunits are encoded by the *PSMB6*, *PSMB7*, and *PSMB5* genes [20]. The proteasome directly influences apoptosis and the cell cycle through degradation of key proteins such as cyclins, caspases, BCL2, and nuclear factor kappa B (NF-κB) [15].

Although both normal and malignant cells rely on proteasome function, cancer cells usually present with dysregulated protein homeostasis and uncontrolled gene transcription [21,22,23,24], rendering them more sensitive to proteasome inhibition [15]. This vulnerability is attributed either to a stronger dependency of highly proliferative cells on the proteasome to remove misfolded proteins, the dependency on NF-κB pathway activation, or pre-existing defects in the cell-cycle and apoptotic pathways that lead to tumorigenesis [15].

Targeting protein degradation has been used as a strategy in cancer therapy since 2003, when bortezomib received approval by the Food and Drug Administration (FDA) for the treatment of relapsed multiple myeloma [25,26]. However, bortezomib has shown some limitations in clinical use, including primary resistance in some patients and limited efficacy in solid tumors [27,28]. Carfilzomib is a second-generation proteasome inhibitor that binds irreversibly and selectively to the β5 subunit of the 26S proteasome, inhibiting its chymotrypsin-like activity [29]. This selective inhibition has been shown for a variety of cellular and in vivo systems, and results in improved specificity and a more favorable toxicity profile compared to bortezomib [29,30,31].

Carfilzomib has already shown promising in vitro effects in several pediatric solid tumors such as neuroblastoma, rhabdomyosarcoma, osteosarcoma, and Ewing sarcoma, as well as pediatric leukemia [32]. Based on these findings, we investigated the therapeutic potential of carfilzomib in HB. We evaluated carfilzomib responses in established HB cell lines as well as patient-derived xenograft (PDX) models. To better recapitulate tumor architecture and micro-environmental conditions, we further established three-dimensional PDX models and assessed their response to carfilzomib treatment.

## 2. Materials and Methods

### 2.1. Public Dataset Analysis

Publicly available transcriptomic datasets were used to compare RNA expression levels of PSMB5, PSMB6, and PSMB7 in normal liver and hepatoblastoma (HB) samples. The datasets GSE133039, GSE104766, and GSE131329 were analyzed. Processed RNA sequencing data were accessed via the R2 genomics analysis and visualization platform (https://hgserver1.amc.nl/).

The global gene expression profiling of RNA sequencing (RNA-seq) for DMSO and carfilzomib-treated cardiomyocytes was obtained from the National Center for Biotechnology Information Gene Expression Omnibus (NCBI-GEO) database with the accession number GSE163102 [33]. In this dataset, cells were treated with DMSO (a vehicle control) or 1 µmol/L carfilzomib (a proteasome inhibitor) for 24 h prior to RNA isolation and sequencing. Expression values were reported as FPKM (fragments per kilobase of transcript per million mapped reads; normalized RNA-seq metric). Gene set enrichment analysis was performed (GSEA) (https://www.gsea-msigdb.org/gsea/index.jsp, accessed on 6 May 2026) with gene sets from the Molecular Signatures Database (MSigDB 2025.1). Enrichment was assessed for Hallmark gene sets (biological processes) and Kyoto Encyclopedia of Genes and Genomes (KEGG) pathways.

Over-representation analysis (ORA; enrichment of predefined functional categories) was conducted using the Enrichr (https://maayanlab.cloud/Enrichr/, accessed on 6 May 2026) based on significantly dysregulated (*p* < 0.05, absolute fold change >1.3) proteins (182 up- and 39 down-regulated) from the proteomics dataset MSV000087350 [33]. This dataset includes quantitative proteomic analysis of human-induced pluripotent stem cell-derived cardiomyocytes treated with 1 µmol/L carfilzomib or DMSO for 24 h. Gene expression data and overall survival data of patients with cervical squamous cell carcinoma (CESC, n = 283), kidney chromophobe renal cell carcinoma (KICH, n = 64), lung adenocarcinoma (LUAD, n = 497), bladder urothelial carcinoma (BLCA, n = 169), head and neck squamous cell carcinoma (HNSC, n = 492), and hepatocellular carcinoma (HCC, n = 365) were retrieved from Human Protein Atlas database v24.0 (https://www.proteinatlas.org/) and used for survival analysis.

### 2.2. Cell Lines

For cell culture experiments, we used 12 patient-derived xenograft (PDX) cell lines, developed from serial PDX transplantation models. The PDX cell lines were purchased from (XenTech, Evry, France) and maintained in advanced DMEM/F12 supplemented with 10% (*v*/*v*) fetal bovine serum, 1% (*v*/*v*) penicillin/streptomycin, 1% (*v*/*v*) L-glutamine (all from Thermo Fisher, Waltham, MA, USA), and the Rho-associated kinase inhibitor Y-27632 (Selleckchem, Chesterbrook, PA, USA) at a final concentration of 20 µM. As healthy controls, we used human dermal fibroblasts HDFa (adult) and HDFn (neonatal), the embryonic kidney cell line HEK293 (all American Type Culture Collection, Manassas, VA, USA), as well as the keratinocyte cell line HaCaT (CLS Cell Lines Service, Eppelheim, Germany). All healthy controls were maintained in DMEM (Life Technologies, Carlsbad, CA, USA) with 10% fetal bovine serum and 1% penicillin/streptomycin. All cell lines were routinely checked for mycoplasma contamination using the mycoplasma PCR detection kit (Applied Biological Materials, Richmond, BC, Canada) and cell identity was monitored by morphological characteristics and the ß-catenin mutation status [8].

### 2.3. Viability Assay

5 × 10^4^ cells/well were seeded in 96-well plates and exposed to 10 increasing concentrations of carfilzomib (Selleckchem, Planegg, Germany), with a 1:3 constant dilution ratio, ranging from 5 nM to 100 µM for 48 h. Cell viability was measured by an MTT assay (3-(4,5-dimethylthiazol-2-yl)-2,5-diphenyltetrazolium bromide) (Sigma-Aldrich, St. Louis, MO, USA) and drug sensitivity was determined by calculating the area under the response curve and half-maximal inhibitory concentrations (IC50) using GraphPad Prism 8 software (GraphPad Software, San Diego, CA, USA).

### 2.4. Proliferation Assay

Short-term growth was detected using a proliferation assay by seeding 1 × 10^5^ cells in a 24-well plate and exposing them to 500 nM carfilzomib or DMSO for 24 h. Proliferating cells were detected by the Click-iT 5-Ethynyl-2′-deoxyuridin (EdU) Imaging Kit (Thermo Fisher) according to the manufacturer’s instructions and fluorescent images were captured by EVOS M7000 (Thermo Fisher). Relative proliferation was calculated by the ratio of the EdU-positive (proliferating) cells to the Hoechst 33342-positive (total) cells. Long-term growth was determined by colony formation assay, by seeding 2 × 10^3^ cells in a 6-well plate and treating them with 25 nM carfilzomib, 100 nM carfilzomib, or DMSO for 10 days. Formed colonies were stained with 0.5% crystal violet (Sigma-Aldrich, St. Louis, MO, USA) in 20% methanol for 2 h. Images of colonies were taken by the GelJet Imager (INTAS, Göttingen, Germany).

### 2.5. Apoptosis Assay

Cells that undergo apoptosis were detected by CellEvent™ Caspase-3/7 Green Detection Reagent (Thermo Fisher) according to the manufacturer’s instructions. Relative apoptosis was determined by referring cells harboring the active substrates of caspase 3 and 7 to the Hoechst-33342-positive (total) cell number. All images were captured using the EVOS M7000 imaging system (Thermo Fisher). Apoptosis rescue experiments were performed by conducting an MTT-based viability assay. The cells were pre-exposed for 4 h to 20 µM of the cell-permeant and irreversible pan-caspase inhibitor Z-VAD-FMK (Benzyloxycarbonyl-Valyl-Alanyl-Aspartyl-Fluoromethylketone) (Bachem AG, Bubendorf, Switzerland) and then treated with DMSO or 500 nM carfilzomib for 48 h.

### 2.6. Life and Death Staining

Decellularised liver matrices (DLMs) were used as scaffolds for three-dimensional (3D) growth and 2 × 10^3^ HB PDX cells were incubated with the DLMs for 14 days in a 24-well plate as previously described [34]. Upon re-population of cancer cells into the DLMs, they were exposed to 500 nM carfilzomib or DMSO for 48 h. Assessment of viability upon treatment was detected by simultaneous staining with 1 µM calcein-AM (BioLegend, San Diego, CA, USA) as a viability marker, 2 μg/mL propidium iodide (PI, Sigma-Aldrich) as a death marker, and 10 μg/mL Hoechst 33342 (Thermo Fisher) as a nuclear counterstain for 20 min at 37 °C. All fluorescent images were captured by the EVOS™ M7000 imaging system (Invitrogen, Carlsbad, CA, USA).

### 2.7. Histology

3D HB models were embedded in 1.5% (*w*/*v*) agarose and then fixed in 4% (*w*/*v*) paraformaldehyde (PFA, Sigma-Aldrich) in PBS (Thermo Fisher) solution for 4 h. All samples were washed with PBS and dehydrated through a series of ethanol washes (50%, 70%, 90% and 100%, 2 × 30 min, respectively). This was followed by incubation in ROTI^®^Histol (Roth, Karlsruhe, Germany) for 30 min in 50% (*v*/*v*) ROTI^®^Histol in paraffin (McCormick Scientific, St. Louis, MO, USA) and for 2 × 60 min in pure paraffin overnight. Samples were embedded in paraffin blocks and cut into sections of 5 µm thickness using a Leica SM2000R microtome (Wetzlar, Germany). Slides were deparaffinized in ROTI^®^Histol and rehydrated using descending ethanol washes. Using Mayer’s Hematoxylin (Roth, Karlsruhe, Germany) and Eosin (Sigma-Aldrich), Hematoxylin and Eosin (H&E) staining was performed.

### 2.8. RNA Expression Analysis

Total RNA was extracted from carfilzomib (500 nM) and DMSO-treated cells, reverse-transcribed using Superscript II, and quantitatively analyzed by real-time PCR as previously described [35]. The following primer pairs (5′ → 3′ orientation) were used: NQO1, GCTGCCATGTATGACAAAGGAC, CCGGTGGATCCCTTGCAGA; TXNRD1, GTTACTTGGGCATCCCTGGTGA, CGCACTCCAAAGCGACATAGGA; SOD1, GGTGTGGCCGATGTGTCTAT, GCTTTTTCATGGACCACCAGT; and TBP GCCCGAAACGCCGAATAT, CCGTGGTTCGTGGCTCTCT. Relative RNA expression was calculated using the ∆∆CT method and expressed as the fold change relative to the corresponding DMSO-treated sample [36].

### 2.9. Reactive Oxygen Species Assay

The reactive oxygen species (ROS) detection assay kit (Abcam, Cambridge, UK) was used to determine cellular ROS levels following the manufacturer’s instructions. 5 × 10^5^ cells were seeded in a 6-well plate and exposed to DMSO or 500 nM carfilzomib for 48 h; they were then stained with permanent 2′-7′-dichlorofluorescin diacetate (DCFDA) dye for 30 min at 37 °C to detect global ROS changes in live cells [37].

In total, 30 µM of exogenous inducer of oxidative stress, tert-butyl hydroperoxide (TBHP), was used as a positive control and added 4 h prior to the DCFDA staining. ROS levels were determined by the EVOS M7000 (Thermo Fisher) imaging system. Detection of carfilzomib response when ROS production was inhibited was performed by the MTT-based viability assay. The cells were pre-exposed for 4 h to 5 mM of ROS scavenger N-acetyl-cysteine (NAC) (Sigma-Aldrich, St. Louis, MO, USA), and then treated with DMSO or 500 nM carfilzomib for 48 h.

### 2.10. Statistical Analysis

Statistical analysis was carried out using Microsoft Excel 2019 (Microsoft Corporation, Redmont, WA, USA) and GraphPad Prism 8.2.1.0 software (GraphPad Software, San Diego, CA, USA).

## 3. Results

### 3.1. Hepatoblastoma Displays Proteasome Enrichment and PSMB5 Overexpression

**Figure 1:**
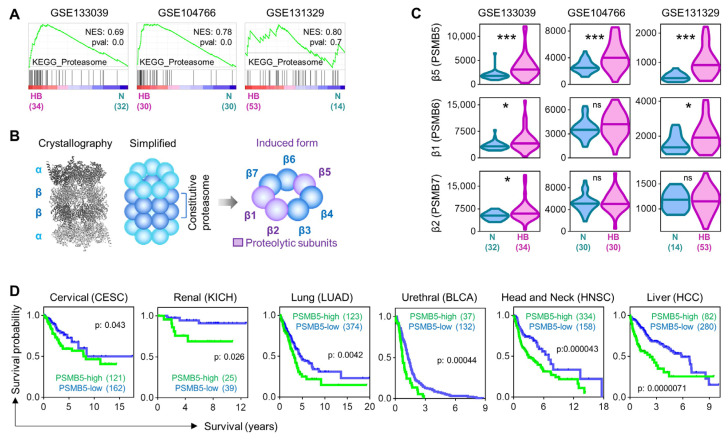
Hepatoblastoma exhibits proteasome enrichment and overexpression of *PSMB5*. (**A**) Enrichment plots displaying the KEGG pathway “proteasome” signatures from publicly available HB datasets GSE133039, GSE104766, and GSE131329. The black vertical lines show the genes coding for proteasome-associated genes, with genes upregulated in hepatoblastoma (HB) to the left. Sample numbers are given in brackets. NES = normalized enrichment score. (**B**) Crystallographic structure of the 20S proteasome with its alpha (α) and beta (ß) subunits obtained from the PoseView tool (left), and simplified versions of the 20S proteasome (middle) and the beta subunits (right). (**C**) RNA expression levels of the *PSMB5*, *PSMB6*, and *PSMB7* genes, obtained from the R2 genomic analysis and visualization platform, are shown in normal liver (N) and HB samples. Fragments per kilo base of transcript per million mapped reads of candidate genes were retrieved from the datasets GSE133039, GSE104766 and GSE131329 (sample number in brackets). The violin plots display distribution of RNA expression, including the median of the groups (horizontal lines). Statistics were calculated using a two-tailed unpaired Student’s *t* test, with ns = not significant, * *p* < 0.05, *** *p* < 0.001. (**D**) Kaplan–Meier curves displaying survival probabilities for indicated cancer types, retrieved from the Human Protein Atlas Database with either high or low PSMB5 expression. The log-rank Mantel–Cox test was used to calculate significance.

To identify potential therapeutic targets for hepatoblastoma (HB), we performed gene set enrichment analysis (GSEA) of three publicly available datasets (GSE133039, GSE104766, and GSE131329). Across all datasets, the KEGG proteasome was consistently enriched in HB samples compared to normal liver tissue (Figure 1A). The 20S core particle in the proteasome includes the proteolytic subunits β1, β2, and β5, encoded by the genes *PSMB6*, *PSMB7* and *PSMB5*, respectively (Figure 1B). In all three datasets, we found *PSMB5* to be significantly upregulated in HB compared to normal liver samples, whereas *PSMB6* and *PSMB7* showed no consistent changes (Figure 1C). As survival data were not available for HB, we examined various adult tumor cohorts stratified by *PSMB5* expression. We found that high *PSMB5* levels were generally associated with poorer survival, with the most marked difference in hepatocellular carcinoma (HCC) patients (Figure 1D). These findings identify *PSMB5* as a consistently overexpressed proteasome subunit in HB and suggest its potential as a prognostic marker and therapeutic target.

### 3.2. Carfilzomib Reduces Cell Viability in HB and PDX Cell Lines

**Figure 2:**
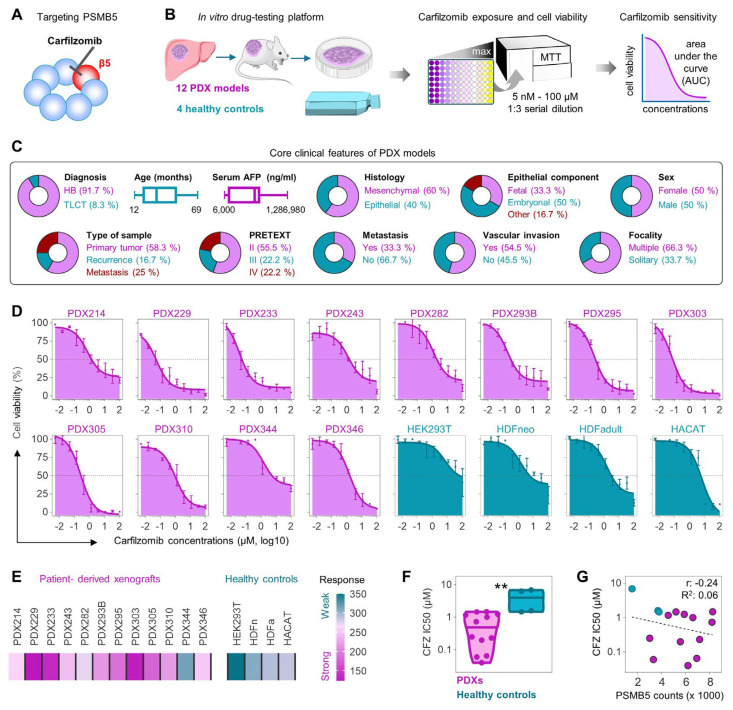
Carfilzomib reduces cell viability in HB and PDX cell lines. (**A**) Illustrative representation of the β5 proteolytic subunit of the proteasome targeted by carfilzomib. (**B**) Schematic display of the in vitro drug testing and determination of drug response. (**C**) Core clinical features of PDX models. Age and AFP distribution are given as a min-to-max floating box plot and vertical lines show the median value. (**D**) Cell viability curves demonstrating carfilzomib sensitivity in HB PDX models (purple) and non-cancerous healthy control cell lines (green). Dashed lines represent the IC50 values. (**E**) A heatmap displaying area under the curve values. (**F**) Violin plots showing carfilzomib sensitivity; each symbol represents the IC50 value of the individual model. (**G**) Correlation between response of hepatoblastoma models towards CFZ, given as half-maximal inhibitory concentrations (IC50) and RNA sequencing-derived PSMB5 expression, with R^2^ correlation coefficients and *p*-values calculated by Pearson’s two-tailed test and linear regressions given as dashed lines. Significance was calculated using a two-tailed unpaired Student’s *t*-test, with ** *p* < 0.01. AFP, alpha-fetoprotein; HB, hepatoblastoma; TLCT, transitional liver cell tumor; PRETEXT, pre-treatment extent of tumor.

Carfilzomib, a selective inhibitor of the β5 proteolytic subunit of the proteasome (Figure 2A) [29], was tested based on our finding that *PSMB5* is the most consistently overexpressed gene across all three HB datasets (see Figure 1C). In order to evaluate the sensitivity of HB cells to carfilzomib, our established in vitro drug-testing platform, consisting of 12 PDX models and four non-cancerous healthy cell lines, was employed (Figure 2B). Our PDX models covered different tumor types and various typical clinical features, thereby providing a representative cohort for drug testing (Figure 2C). The assessment of MTT-based drug response resulted in a clear, dose-dependent reduction in cell viability (Figure 2D). Moreover, decreased sensitivity towards carfilzomib was observed in healthy controls, as indicated by elevated areas under the curve (mean AUC for PDXs: 209.9 vs. mean AUC of controls: 308.9) (Figure 2E) and higher IC50 values (mean IC50 for PDXs: 0.67 µM vs. mean IC50 of controls: 3.77 µM) (Figure 2F). Importantly, a negative correlation between carfilzomib response and PSMB5 expression was detected in our screening platform (Figure 2G).

### 3.3. Carfilzomib Effectively Inhibits Growth and Induces Apoptosis in HB Cells

**Figure 3:**
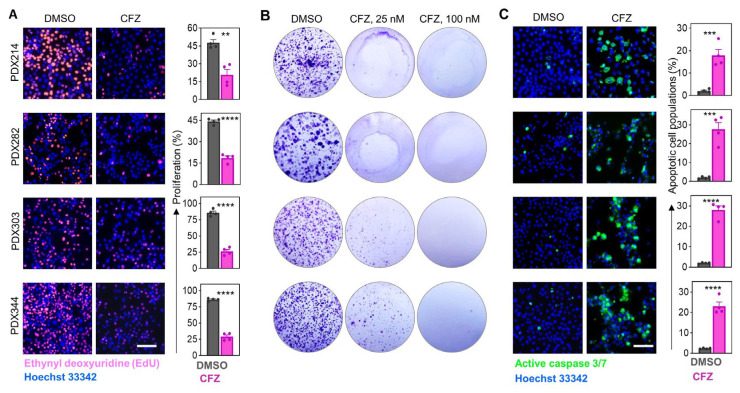
Carfilzomib (CFZ) effectively inhibits growth and induces apoptosis in HB cells. (**A**) Proliferating cells were detected by EdU staining (red) and quantified in relation to Hoechst 33342-stained nuclei (blue). PDX cells were exposed to DMSO or 500 nM carfilzomib for 24 h. (**B**) Long-term growth of the PDX cells was detected by colony formation assays. Cells were exposed to DMSO or 25 nM or 100 nM carfilzomib for 10 days, and colonies were stained with crystal violet. (**C**) Apoptotic cells were detected in HB PDX cells, which were exposed to DMSO or 500 nM carfilzomib for 24 h by staining the active caspase 3/7 substrate (green) and quantifying it in relation to Hoechst-33342-stained nuclei (blue). All bar graphs show the mean ± SEM of two independent experiments in duplicates. Statistics were calculated using a two-tailed unpaired Student’s *t* test, with ** *p* < 0.01, *** *p* < 0.001, **** *p* < 0.0001. All scale bars represent 100 µm.

To explore the mechanisms underlying the reduction in cell viability after treatment with carfilzomib, we assessed proliferation and apoptosis in HB PDX cells. Carfilzomib significantly inhibited short-term cell proliferation, as shown by EdU incorporation after 24 h (Figure 3A). Long-term growth was also strongly impaired, given the substantial reduction in colony formation after 10 days of treatment with 25 nM carfilzomib, while 100 nM almost completely abolished colony formation (Figure 3B). Correspondingly, assessment of apoptosis using caspase 3/7 staining revealed a significant increase in apoptotic cells in carfilzomib-treated HB PDX cell lines compared to controls (Figure 3C), indicating that carfilzomib inhibits HB cell growth through both proliferation arrest and induction of apoptosis.

### 3.4. Carfilzomib Inhibits Growth of Three-Dimensional HB Models

**Figure 4:**
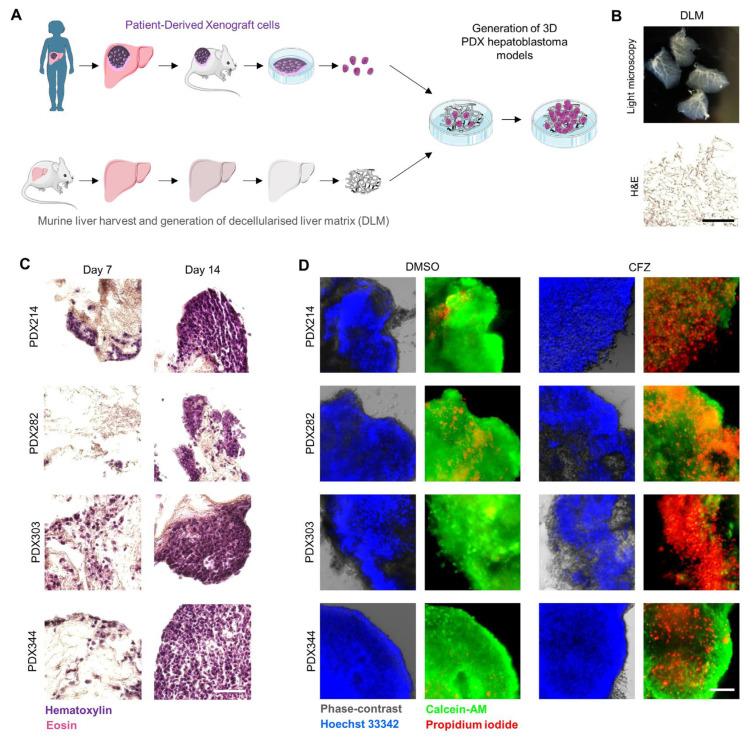
Carfilzomib (CFZ) inhibits growth of three-dimensional PDX models. (**A**) Schematic overview depicting the establishment of 3D PDX hepatoblastoma models. (**B**) Light microscopic photograph (top) and a Hematoxylin/Eosin (HE)-stained section (bottom) of decellularized liver matrices (DLM) that served as a liver-specific scaffold. Scale bar is 100 µm. (**C**) HE stains of 3D PDX models after 7 and 14 days of cultivation. Scale bar is 100 µm. (**D**) Fluorescent images of life (calcein-AM, green)/death (propidium iodide, red) staining of DLMs populated with HB PDX cells (counterstained with Hoechst 33342, blue) for 14 days, and treated with DMSO or 500 nM carfilzomib for 48 h. Scale bar is 100 μm.

To simulate more physiologically relevant conditions, we generated three-dimensional (3D) HB models using PDX cell lines. PDX cells were seeded onto decellularized liver matrices (DLMs), providing a natural extracellular scaffold that supports cell adhesion, organization, and tissue-specific interactions (Figure 4A,B). The populated scaffolds were then cultured under appropriate conditions until fully formed 3D structures were established, mimicking the architectural and spatial features of in vivo tumors (Figure 4C). To assess the impact of proteasome inhibition in this model, 3D PDX constructs were treated with carfilzomib. Life/death staining showed a strong increase in dead cells in carfilzomib-treated 3D models compared to untreated models, demonstrating that the drug effectively induces cytotoxicity even in a complex, tissue-like environment (Figure 4D). These results highlight the utility of 3D PDX models for evaluating therapeutic responses and suggest that carfilzomib retains efficacy in conditions that closely mimic the in vivo tumor architecture.

### 3.5. Carfilzomib Prevents HB Cell Growth in a ROS-Dependent Manner

**Figure 5:**
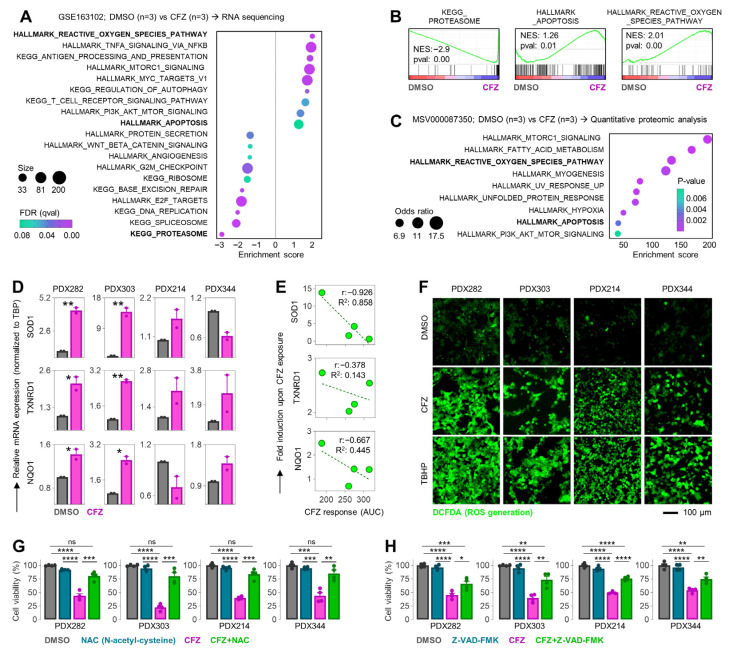
Carfilzomib (CFZ) prevents HB cell growth through ROS generation. (**A**) Bubble plot demonstrates the list of enriched pathways upon Gene Set Enrichment Analysis (GSEA) of RNA sequencing results from the GSE163102 dataset, which includes DMSO- (n = 3) and CFZ-treated (n = 3) cardiomyocytes. Bubble size represents the genes that are upregulated within the pathway; green-to-purple color scale indicates the false discovery rate (FDR). (**B**) Enrichment plots for KEGG proteasome, HALLMARK apoptosis and HALLMARK reactive oxygen species (ROS) are displayed. (**C**) The bubble plot, derived from significantly dysregulated proteins from the MSV000087350 dataset, displays enriched pathways. A total of 182 up- and 39 down-regulated (*p* < 0.05, absolute fold change >1.3) proteins were analyzed by Enrichr, where bubble size represents the odds ratio and green-to-purple color scale indicates *p*-values. (**D**) Quantitative PCR-based mRNA expression levels of *SOD1*, *TRNXD1*, and *NQO1* genes upon carfilzomib exposure are demonstrated. Results were normalized to the housekeeping gene TBP. Bar graphs represent two independent experiments. The significance was calculated using a two-tailed unpaired Student’s *t* test, with * *p* < 0.05, and ** *p* < 0.01. (**E**) Correlation of carfilzomib response and fold induction of *SOD1*, *TRNXD1*, and *NQO1* genes was calculated by a two-tailed Pearson test. (**F**) Cellular ROS levels were detected by DCFDA staining (green) in PDX models treated with DMSO or 500 nM carfilzomib. (**G**) Cell viability of PDX models that were pre-treated for 4 h with N-acetyl-cystein (NAC), then exposed to DMSO or 500 nM carfilzomib for 48 h. (**H**) Cell viability of PDX models that were pre-treated for 4 h with Benzyloxycarbonyl-Valyl-Alanyl-Aspartyl-Fluoromethylketone (Z-VAD-FMK), then exposed to DMSO or 500 nM carfilzomib for 48 h. The significance for all graphs was calculated using a two-tailed unpaired Student’s *t* test, with ns = not significant, * *p* < 0.05, ** *p* < 0.01, *** *p* < 0.001, **** *p* < 0.0001.

Carfilzomib not only reduces cell viability, cell proliferation, and colony formation, but also increases apoptosis in HB PDX cell lines. To explore the molecular consequences of treatment in more detail, we analyzed the GSE163102 dataset, which includes the differential transcriptomic profiles of three carfilzomib-treated cardiomyocytes in comparison to three DMSO controls [33]. As expected, the KEGG proteasome was strongly down-regulated, while the hallmarks of apoptosis and reactive oxygen species (ROS) showed strong positive enrichment in carfilzomib-treated cells (Figure 5A). Enrichment plots for all three pathways further highlighted the differential activity between DMSO- and carfilzomib-exposed cardiomyocytes (Figure 5B). More importantly, enriched pathways generated using significantly dysregulated proteins from quantitative proteomic analysis of carfilzomib-exposed cardiomyocytes showed enriched HALLMARK apoptosis and reactive oxygen species (ROS) pathways (Figure 5C). To validate these effects in HB, we measured the mRNA expression levels of key ROS pathway genes (*SOD1*, *TRNXD1*, and *NQO1*) using quantitative PCR, normalizing this to the housekeeping gene *TBP*. The HB PDX cell lines PDX282 and PDX303 showed significantly higher mRNA expression of *SOD1*, *TRNXD1*, and *NQO1* compared to controls, whereas no significant induction of ROS pathway genes was observed in PDX214 and PDX344 (Figure 5D). When we plotted the induction of ROS pathway genes against the corresponding carfilzomib response in each cell line, we saw a strong negative correlation for *SOD1* mRNA expression with carfilzomib response, suggesting that ROS pathway activation contributes to the therapeutic effect of carfilzomib (Figure 5E). Accordingly, using a ROS detection assay, we found higher ROS generation in carfilzomib-treated cell lines compared to controls, with ROS levels comparable to cells treated with TBHP: an exogenous inducer of oxidative stress used as a positive control (Figure 5F).

As a proof of concept study, we performed cell viability assays in PDX cell lines after exposure to DMSO (control), the ROS scavenger N-acetyl-cystein (NAC), carfilzomib, or a combination of NAC and carfilzomib. Carfilzomib alone led to a significant reduction in cell viability compared to controls and cells exposed only to NAC. Importantly, pre-treatment with NAC abolished carfilzomib-induced cytotoxicity, demonstrating that ROS generation is important for the antitumor effect of carfilzomib in HB PDX cells (Figure 5G). Next, we investigated the mechanism of ROS-induced cell death upon carfilzomib exposure. The rescue experiment with the pan-caspase inhibitor Z-VAD-FMK and carfilzomib clearly demonstrated that growth inhibition by carfilzomib is mediated by ROS-induced apoptosis (Figure 5H).

Together, these results indicate that carfilzomib suppresses HB cell growth through a dual mechanism of proteasome inhibition and ROS-mediated apoptosis, highlighting oxidative stress as a key contributor to its therapeutic efficacy.

## 4. Discussion

Hepatoblastoma (HB) remains a challenging disease for targeted therapy development, as no single actionable genetic target has been identified. Proteasome inhibition has emerged as an effective therapeutic approach across a variety of cancer types by interfering with several key cellular processes. An enrichment of proteasome-related gene sets in HB prompted us to investigate proteasome inhibition as a potential novel therapy. Our findings demonstrate that selective targeting of the HB-upregulated β5 proteolytic subunit of the proteasome by carfilzomib induces dose-dependent reduction in viability, proliferation, and clonogenicity, accompanied by strong apoptosis across multiple HB PDX models, including physiologically relevant three-dimensional liver matrix cultures. Mechanistically, these effects are mediated by proteasome inhibition-induced oxidative stress, as ROS pathway activation is correlated with drug sensitivity, and ROS scavenging completely eliminates carfilzomib cytotoxicity.

The rationale for targeting the proteasome in HB was further supported by our observation that proteasome-related gene sets were significantly enriched in HB transcriptomic datasets. In addition, we detected elevated *PSMB5* expression in tumor tissue compared to normal liver, but not uniformly for other proteasomal genes such as *PSMB6* and *PSMB7*. *PSMB5* plays a tumor-promoting role in various human cancers, influencing growth, metastasis, drug resistance, and tumor development [38,39]. *PSMB5* codes for the β5 subunit of the proteasome, which mediates its chymotrypsin-like activity [22] and represents the primary molecular target of the proteasome inhibitor carfilzomib [29,30,31]. Overexpression of *PSMB5* has been reported in a broad range of malignancies, including esophageal, renal, urethral, breast, prostate, lung cancers, and hepatocellular carcinoma (HCC), and is frequently associated with poor prognosis [38,39,40,41,42]. In line with this, we found that high *PSMB5* levels correlate with poorer survival in many cancer types, most markedly in HCC. Whether an overexpression of *PSMB5* in HB also implies a poorer prognosis remains unclear. More importantly, *PSMB5* overexpression has also been linked to resistance to the first-generation proteasome inhibitor bortezomib [39,41,42,43]. Unlike bortezomib, a reversible proteasome inhibitor that has previously shown efficacy in HB cells and xenografts [13,44], carfilzomib irreversibly inhibits the proteasome with higher specificity and less off-target activity [45,46]. Moreover, studies in childhood leukemia have shown that carfilzomib is associated with fewer adverse drug reactions and may overcome resistance to bortezomib [47,48]. Although response rates varied across cell lines and models, our results show that proteasome inhibition by carfilzomib was effective in the investigated PDX cell lines, including physiologically relevant three-dimensional liver matrix cultures.

Consistent with our results in HB, carfilzomib has also been shown to inhibit proliferation and induce apoptosis in multiple tumor types, including HCC and pediatric solid tumors such as Ewing’s sarcoma, osteosarcoma, neuroblastoma, and rhabdomyosarcoma [32,49]. In HCC, carfilzomib led to cell cycle arrest in the G2/M phase with a reduction in cyclin-dependent kinases, but also an increase in ROS production with a subsequent increase in endoplasmatic reticulum (ER) stress and JNK/p38 MAPK-mediated apoptosis [50]. This increase in ROS was attributed to carfilzomib-induced mitochondrial dysfunction [50]. For leukemia cell lines, the induction of ER stress leads to a similar cell cycle arrest in the G2/M phase and an induction of intrinsic and extrinsic apoptosis via the ubiquitin proteasome pathway; an induction of autophagy was also detected [51,52]. Carfilzomib-induced autophagy, however, has been described to promote cell survival in HNSC, and a simultaneous inhibition of autophagy might be necessary [53].

Here, we identified a previously unrecognized mechanism by which carfilzomib induces apoptosis in HB cells. Our data indicate that carfilzomib suppresses HB cell growth through a reactive oxygen species (ROS)-mediated mechanism, characterized by increased ROS levels and induction of ROS-responsive genes. Although we identified ROS signaling as a target of carfilzomib treatment in cardiomyocytes, this concept was also confirmed in HB models, underscoring a general mode of action across different cell types. However, further transcriptomic and proteomic studies are needed to better understand the effects of carfilzomib on HB cells. Carfilzomib treatment was associated with down-regulation of *PSMB5* and upregulation of *SOD1*, a pattern previously observed in liver cells subjected to oxidative stress [54]. Collectively, these studies highlight the pleiotropic effects of carfilzomib on intracellular signaling networks, emphasizing that only a fraction of its therapeutic potential has been fully characterized to date, suggesting additional, yet unexplored, therapeutic implications in HB. Recognizing that preclinical pharmacologic testing in conventional two-dimensional cell cultures may overestimate therapeutic efficacy [34,55], we evaluated carfilzomib in three-dimensional PDX models to better mimic in vivo conditions. More importantly, the antitumor effects observed in conventional HB cell culture were reproducibly maintained in these physiologically relevant models, strengthening the translational relevance of our findings and supporting the robustness of proteasome inhibition as a therapeutic strategy in HB.

Translating these findings into the clinical setting remains limited due to a lack of systematic evaluation. Nevertheless, a recent phase 1 trial investigating carfilzomib in combination with etoposide and cyclophosphamide in pediatric patients with refractory leukemia and relapsed solid tumors included three patients with relapsed HB, who exhibited survival times of 120 days, 280 days, and over 2000 days [56]. Although febrile neutropenia was the most common severe adverse event occurring in 50% of patients, these results provide early clinical evidence supporting the feasibility of carfilzomib-based regimens in the pediatric population [56].

A limitation of the use of proteasome inhibitors in the treatment of HB includes off-target effects, treatment-related toxicities, and acquired drug resistance, as previously described for bortezomib [19]. There is currently limited knowledge on the influence of carfilzomib on the developing body of a child, which raises concerns about long-term safety and tolerability in children. The existing literature positions proteasome inhibition primarily as an adjuvant to chemotherapy, enhancing the cytotoxic effects of standard treatment regimens [15]. However, in HB, it must be considered that mutational burden and tumor heterogeneity increase substantially after chemotherapy. Consequently, if targeted therapies are to be effective, their optimal positioning within treatment schedules may be in the first-line setting, prior to the acquisition of additional genetic complexity. Addressing these questions will require further mechanistic studies, extended preclinical models, and carefully designed clinical trials.

## 5. Conclusions

In conclusion, our findings establish carfilzomib-mediated proteasome inhibition as a potential antitumor strategy in HB, with consistent efficacy across conventional cell culture and physiologically relevant three-dimensional models. Mechanistically, carfilzomib was associated with the down-regulation of *PSMB5* expression and an induction of ROS signaling, ultimately leading to apoptosis. Together, these results provide a strong rationale for further preclinical optimization and clinical investigation of carfilzomib-based regimens, particularly in combination with standard chemotherapy, to improve therapeutic options for patients with HB.

## Data Availability

The original contributions presented in this study are included in the article. Further inquiries can be directed to the corresponding author.

## Abbreviations

The following abbreviations are used in this manuscript:

HB	Hepatoblastoma
CFZ	Carfilzomib
PDX	Patient-derived xenograft
ROS	Reactive oxygen species
NF-κB	Nuclear factor kappa B
FDA	Food and Drug Administration
GSEA	Gene set enrichment analysis
NCBI-GEO	National center for biotechnology information gene expression omnibus
CESC	Cervical squamous cell carcinoma
KICH	Kidney chromophobe renal cell carcinoma
LUAD	Lung adenocarcinoma
BLCA	Bladder urothelial carcinoma
HNSC	Head and neck squamous cell carcinoma
HCC	Hepatocellular carcinoma
HDF	Human dermal fibroblasts
MTT	3-(4,5-dimethylthiazol-2-yl)-2,5-diphenyltetrazolium bromide
DLM	Decellularised liver matrix
PFA	Paraformaldehyde
NAC	N-acetyl-cystein
NES	Normalized enrichment score
EdU	5-Ethynyl-2′-deoxyuridin
Z-VAD-FMK	Benzyloxycarbonyl-Valyl-Alanyl-Aspartyl-Fluoromethylketone
PRETEXT	Pre-treatment extent of tumor
AFP	Alpha-feto-protein
TLCT	Transitional liver cell tumor
